# Supplementary material for: Genomic Characterization of a Clinical NDM-1-Producing Klebsiella michiganensis from Brazil
Source: Microorganisms. 2024 Jul 12;12(7):1408. doi: 10.3390/microorganisms12071408 (PMC11278599; doi:10.3390/microorganisms12071408)

**Supplementary Figure S1** – Heatmap visualization of Average Nucleotide Identity between the *Klebsiella* spp. genomes available in the NCBI database. A color key located in the upper left corner represents the percentage of identity between the analyzed genomes. The greater the percentage of identity between the genomes, the warmer the color. The clustering of ID_1060/21 with *K. michiganensis* genomes is observed, as is its distance from other species of the *K. oxytoca* complex. The database used to construct this figure is presented in Supplementary Table S1.

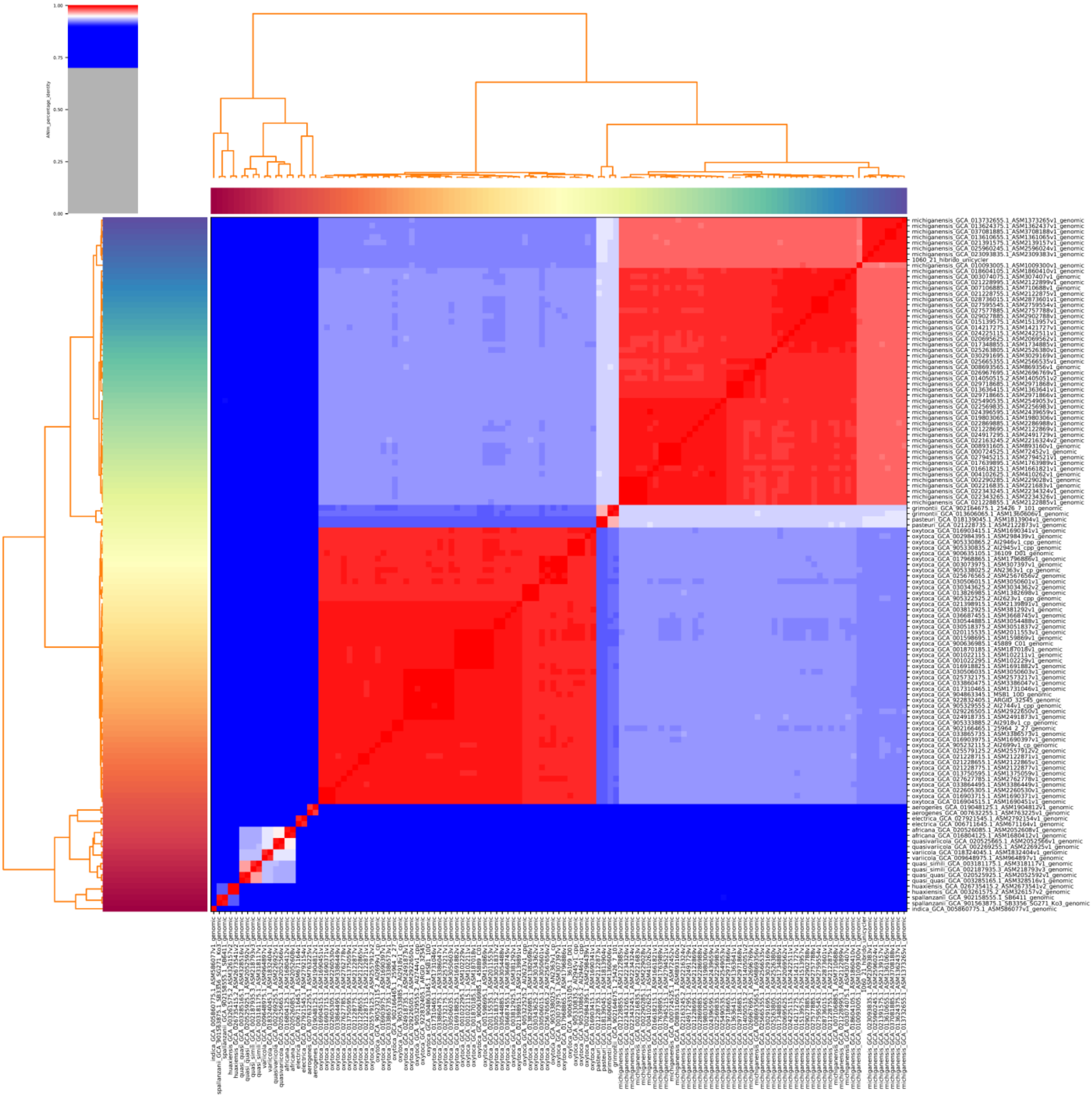

**Supplementary Figure S2** – S1-PFGE of ID_1060/21 (lane 1) and transconjugant *E. coli* J53 with a plasmid of ID_1060/21 (lane 2). It is observed that the wild type of the sample has three plasmids and one of them was transferred to the recipient *E. coli* J53. The plasmid sizes in the wild strain, from top to bottom, according to Bionumerics, are 163.49 kb, 105.91 kb, and 50.52 kb. (M—marker H9812; 1—wild sample ID_1060/21; 2—recipient *E. coli* J53).

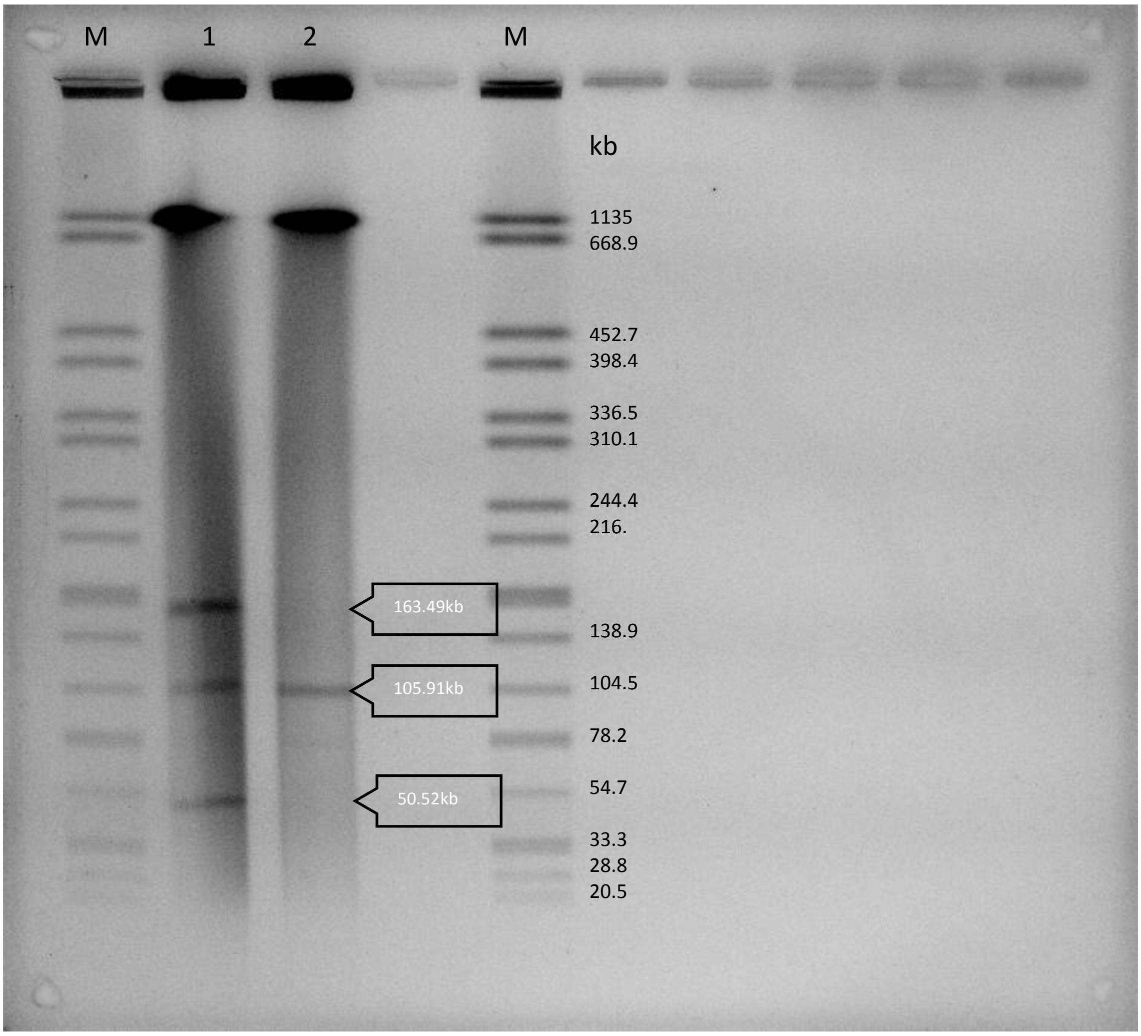

Supplement: Supplementary file 1 [file microorganisms-12-01408-s001.zip › supplementary Figures S1 and S2.pdf]
